# Biocompatibility of a novel directly printed orthodontic aligner at variable material thicknesses

**DOI:** 10.1038/s41598-026-62896-3

**Published:** 2026-07-29

**Authors:** Maximilian Bleilöb, Claudia Welte-Jzyk, Vanessa Knode, Björn Ludwig, Julia Bulski, Christina Erbe

**Affiliations:** 1 Department of Orthodontics and Dentofacial Orthopedics, University Medical Center of the Johannes Gutenberg- University Mainz, Augustusplatz 2, 55131 Mainz, Germany; 2 Department of Orthodontics, University of Homburg, Saar, Germany; 3 Private Practice of Orthodontics, Am Bahnhof 54, 56841 Traben-Trarbach, Germany

**Keywords:** Digital orthodontics, 3D printed aligners, 3D printing, Clear aligner, Aligner therapy, Direct printed aligners, Biocompatibility, Cytotoxicity, Resin, Biotechnology, Health care, Materials science, Medical research

## Abstract

Direct printed aligners (DPAs) represent a revolution in aligner orthodontics, enabling in-office fabrication of customized removable orthodontic appliances and precise, region-specific thickness modulation within a single aligner. Accordingly, this study assessed whether the manufacturer’s standard 20-minute UV post-cure is adequate to ensure biocompatibility of DPAs made from LuxCreo DCA resin (LuxCreo Inc., Chicago, IL, USA) as appliance thickness increases up to 6 mm. Specimen discs (Ø 10 mm) of varying thickness (0.5, 1, 2, 4, and 6 mm) were 3D printed and post-processed according to the LuxAlign (LuxCreo Inc., Chicago, IL, USA) closed end-to-end workflow. Thickness was verified by digital caliper and biocompatibility was assessed using the AlamarBlue assay on human gingival fibroblasts. Printed specimens closely matched nominal dimensions and cell viability always remained above 70%, the ISO 10993-5 threshold for non-cytotoxicity. No significant differences were observed between the standard 0.5 mm and thicker groups (1–6 mm), indicating that increased thickness did not further impair biocompatibility. These findings demonstrate that the standardized LuxAlign workflow, including a 20-minute UV-curing, produces dimensionally accurate, non-cytotoxic appliances from LuxCreo DCA resin up to 6 mm thickness, supporting safe clinical use without extending post-curing.

## Introduction

In contemporary orthodontics, the growing demand for highly aesthetic, hygienic, and comfortable treatment options has driven rapid advancements in clear aligner therapy^1–5^. First conceptualized by Kesling in 1945 as an adjunct for the finishing phase following fixed appliance treatment, clear aligner therapy has evolved considerably over the past three decades^6^. A key milestone driving this development was the introduction of Invisalign (Align Technology, Santa Clara, CA, USA) in 1997^6^. Thereafter, an increasing number of aligner manufacturers have introduced new technologies and materials, expanding the clinical spectrum of clear aligner therapy to include even highly complex malocclusions^1,7^.

In recent years, in-office direct-to-print aligners (DPAs) have emerged as an alternative to conventionally thermoformed aligners^8^. DPAs made from Graphy’s resin Tera Harz TC-85 (Graphy Inc, Seoul, South Korea) were the first to receive CE certification as well as FDA approval^9^. In 2024, Graphy launched an updated and modified version of its resin, Tera Harz TA-28 (Graphy Inc, Seoul, South Korea)^10^. LuxCreo DCA resin (LuxCreo Inc., Chicago, IL, USA) represents another very promising base product having obtained CE certification and FDA clearance for their DPAs^11^. Unlike Graphy’s aligner resins, which are not strictly integrated into a closed end-to-end workflow, LuxAlign (LuxCreo Inc., Chicago, IL, USA) was introduced in 2023 as a fully proprietary solution encompassing resin, software, 3D printer, and curing unit^11^.

DPAs are considered a breakthrough in clear aligner therapy owing to their unique biomechanical and digital advantages compared to their thermoformed counterparts^8,10,12–17^. These benefits particularly include the ability to vary material thickness within the same aligner, allowing the digital design and subsequent manufacture of an almost limitless number of different removable orthodontic appliances^8,10^. For example, this also allows the fabrication of customized functional appliances such as the Twin-Block facilitating Class II treatment^8,10^.

Nevertheless, the introduction of novel materials into clinical practice must always be accompanied by rigorous biocompatibility assessment and independent scientific validation to ensure biological safety for clinical use. Safeguarding patient health must without exception remain the primary obligation for every orthodontist.

While the biocompatibility of DPAs manufactured from both TC-85 and TA-28 resins has been demonstrated in several studies in accordance with ISO standards, even at material thicknesses of up to 6 mm, the biocompatibility of DPAs produced from LuxCreo’s DCA resin (LuxCreo Inc., Chicago, IL, USA) has not yet been independently evaluated^10,18–21^. The present study was designed to evaluate the biocompatibility of DPAs manufactured from LuxCreo’s aligner DCA resin (LuxCreo Inc., Chicago, IL, USA), with a particular focus on whether increasing material thickness above the common clear aligner thickness of 0.5–1 mm, compromises biocompatibility when the duration of UV curing during post-processing is not extended beyond the standard production protocol^22^. This assumption is based on our concern that increased material thickness may result in incomplete polymerization of inner material layers and consequently increased release of residual monomers.

To address this aim, we tested the following null hypotheses:

1. DPAs with a standard thickness of 0.5–1 mm fabricated from LuxCreo’s DCA resin (LuxCreo Inc., Chicago, IL, USA) are biocompatible when manufactured in accordance with the manufacturer’s guidelines.
2. The increase in layer thickness of DPAs made from LuxCreo’s DCA resin (LuxCreo Inc., Chicago, IL, USA) does not require prolongued UV-curing during post-processing to guarantee biocompatibility.
3. Digitally designed specimens, made from LuxCreo’s DCA resin (LuxCreo Inc., Chicago, IL, USA), can be 3D printed to a high degree of accuracy.

## Methods

### Digital design, 3D-printing and post-processing of the specimens

Circular specimens with a diameter of 1 cm were digitally designed using LuxDesign software (Version V2.4.3.60413, LuxCreo Inc., Chicago, IL, USA, https://luxcreo.com) in five different thicknesses (0.5, 1, 2, 4, and 6 mm), with eight specimens produced per thickness in each trial (*n* = 8). The designs were then processed in LuxFlow slicing software (Version V1.5.2.60529, LuxCreo Inc., Chicago, IL, USA, https://luxcreo.com) and printed with the iLux Pro Dental 3D printer (LuxCreo Inc., Chicago, IL, USA) using DCA resin (LuxCreo Inc., Chicago, IL, USA) at a layer thickness of 0.1 mm. After printing, the specimens were washed in 99% isopropanol for 8 min using the iLuxWash Dental system (LuxCreo Inc., Chicago, IL, USA). Post-curing included heat treatment in the LuxOven Pro (LuxCreo Inc., Chicago, IL, USA) at 130 °C for 10 min, followed by UV curing for 20 min without flipping in the iLuxCure Pro (LuxCreo Inc., Chicago, IL, USA). Finally, supports were manually removed and the specimen edges were smoothed using a hand-held buffing wheel operating at 50,000 RPM with 400-grit sandpaper.

Specimen thickness (*n* = 14) was manually measured at the centre of each specimen by a single investigator using a digital caliper (Absolute Digimatic, Mitutoyo, Japan) to assess the dimensional accuracy of the printing process. Measurements were obtained from 14 specimens derived from three independent printing processes. The measurements were carried out only once per specimen and were not repeated.

### Cell culture

Human gingival fibroblasts (HGFs; CLS Cell Lines Service GmbH, Eppelheim, Germany) were cultured in Dulbecco’s modified Eagle medium (DMEM; Thermo Fisher Scientific, Carlsbad, CA, USA) supplemented with 4.5 g/L glucose, 10% fetal bovine serum (Thermo Fisher Scientific, Carlsbad, CA, USA), 100 U/mL penicillin, 100 µg/mL streptomycin, and 50 mg/L L-ascorbic acid (Sigma-Aldrich, Steinheim, Germany). Cells were maintained at 37 °C in a humidified atmosphere of 5% CO₂ and 95% air. Passages four to ten were used for all experiments. For cell detachment, Accutase (Sigma-Aldrich Chemie GmbH, Steinheim, Germany) was utilized. Cells were counted and seeded at a density of 3000 cells per well in 96-well culture plates (Greiner Bio-One, Frickenhausen, Germany) containing 100 µL of medium per well, and were allowed to adhere to the cell bottom for 24 h.

### Specimen testing

**Fig. 1:**
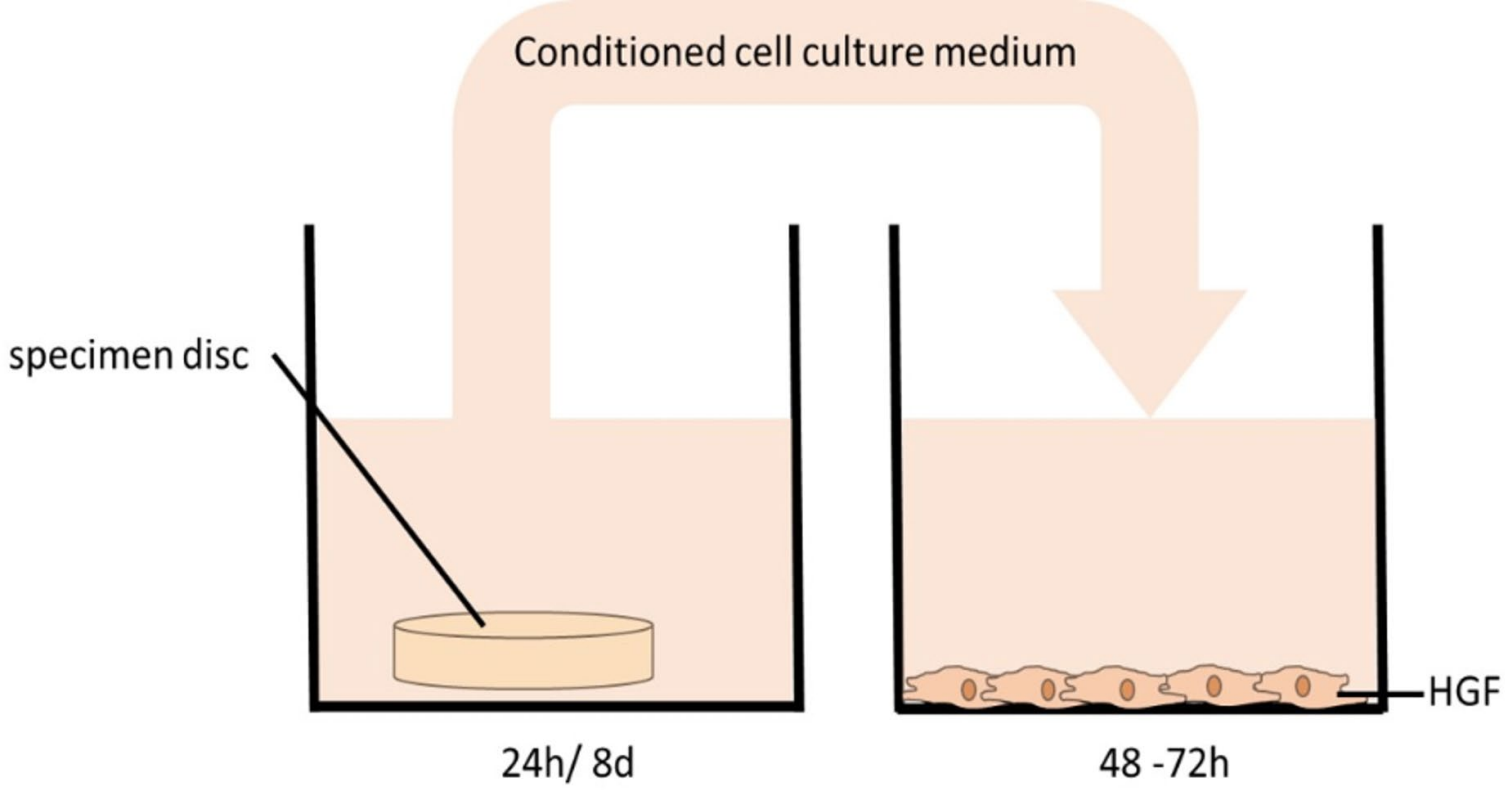
Schematic representation of the experimental workflow used to evaluate the influence of LuxCreo’s DCA aligner resin (LuxCreo Inc., Chicago, IL, USA) on human gingival fibroblasts (HGFs).

To simulate intraoral exposure during active orthodontic treatment, two separate experimental setups were conducted. In the first, specimens were immersed in culture medium under intermittent shaking for 24 h, in accordance with International Organization for Standardization (ISO) Standard 10993. In the second setup, specimens were incubated for 8 days, reflecting the typical clinical wearing time of a clear aligner, which is typically replaced on a weekly basis (Fig. 1). Following each incubation period, the conditioned medium (*n* ≥ 8 per thickness group and experimental trial, with two technical replicates per test specimen) was collected and transferred to 96-well plates containing adherent HGFs. Cell proliferation was continuously tracked using the Incucyte live-cell imaging system (Sartorius, Göttingen, Germany), which allowed real-time monitoring of confluence. In addition, cell viability was determined with the AlamarBlue assay (Thermo Fisher Scientific, Waltham, MA, USA) once cultures had reached approximately 70–80% confluence, typically after 48–72 h (Fig. 1).

### AlamarBlue cell viability assay

The viability of HGFs was evaluated using the AlamarBlue assay (Thermo Fisher Scientific, Waltham, MA, USA), a Resazurin-based method that measures the metabolic activity of living cells. Resazurin is taken up by viable cells and enzymatically reduced to Resorufin, which is red and highly fluorescent, allowing quantitative assessment of cell proliferation and viability. For the assay, AlamarBlue reagent was added directly to the culture medium at a 1:10 dilution. After an incubation period of 3–4 h, fluorescence was recorded with a microplate reader (excitation 560 nm, emission 600 nm; VersaMax Microplate Reader, Molecular Devices, Sunnyvale, CA, USA). Fluorescence values were normalized against untreated controls, which were defined as 100%. Statistical analysis was performed on the mean values obtained. Following ISO 10993-5 guidelines, a decrease in viability exceeding 30% was classified as cytotoxic.

### Statistical analysis

Statistical analysis was performed using one-way ANOVA with Tukey’s post-hoc test, assuming a normal distribution as assessed by the Kolmogorov–Smirnov test. Descriptive statistics, such as mean with standard deviation/standard error of the mean and confidence intervals, were calculated using GraphPad Prism (Version 10.2.0, GraphPad Software, Boston, MA, USA, https://www.graphpad.com).

## Results

### Accuracy of specimen discs

**Fig. 2:**
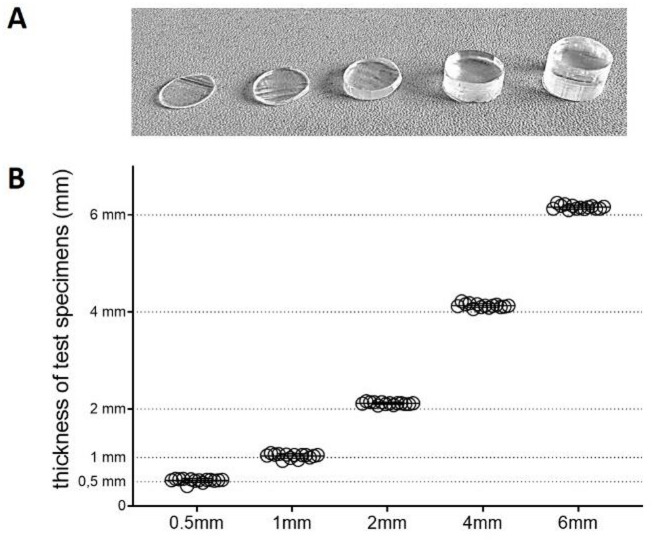
**A** Test specimens printed with LuxCreo’s DCA aligner resin (LuxCreo Inc., Chicago, IL, USA) **B** Measured thicknesses of specimens (*n* = 14) from three independent printing processes, printed at nominal values of 0.5, 1, 2, 4, and 6 mm using LuxCreo’s DCA aligner resin (LuxCreo Inc., Chicago, IL, USA). Post-processing was performed according to the LuxAlign closed end-to-end system (LuxCreo Inc., Chicago, IL, USA), including 20 min of UV curing.

Specimen dimensions were measured manually with a digital caliper, confirming a diameter of 1 cm and accurate thicknesses of 0.5, 1, 2, 4, and 6 mm (Fig. 2).

### Cell viability and proliferation

**Fig. 3:**
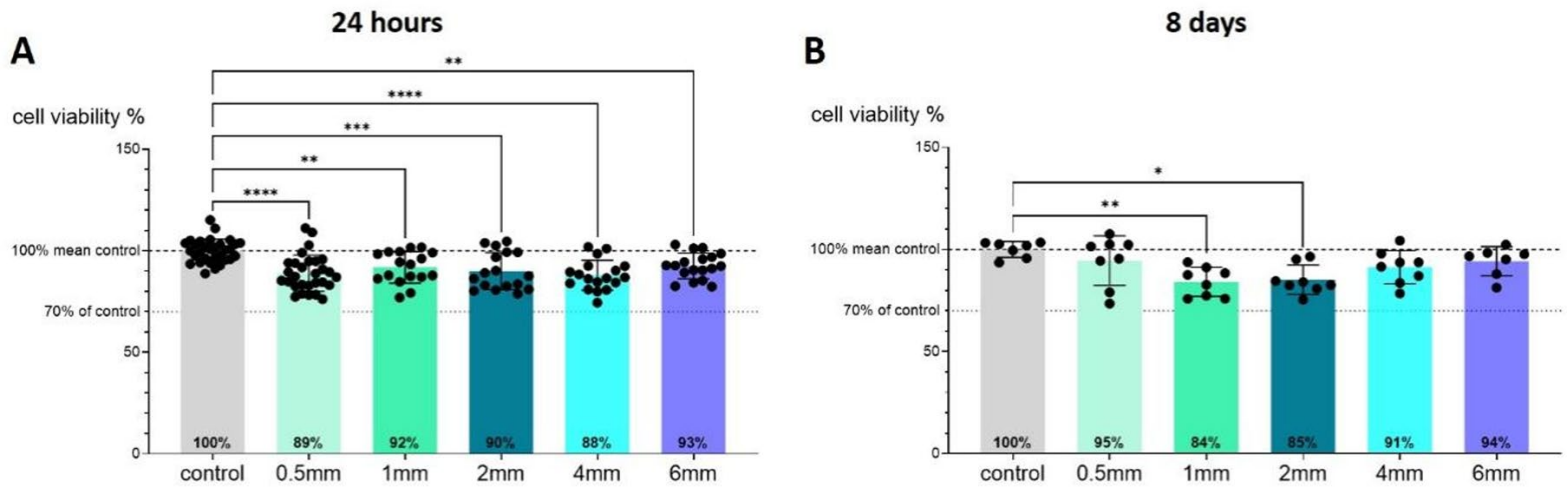
Cell viability of HGF after culture for 24 h (A) and 8 days (B) in media conditioned with LuxCreo test specimens in different thicknesses (0.5, 1, 2, 4, 6 mm). Cell viability of the individual thickness groups was compared with the control (**p < 0.01; ***p < 0.001; ****p < 0.0001). No statistically significant differences in cell viability were observed between the individual thickness groups. Descriptive statistics and the results of the normality test are presented in Table 1.

Although cell viability was significantly reduced compared with the control in all thickness groups, values consistently remained above 70%, the ISO 10993-5 threshold for cytotoxicity, after both 24-hour and 8-day exposure periods (Fig. 3; Table 1). Importantly, no significant differences were detected between the standard thickness of 0.5 mm and the increased thicknesses of 1–6 mm, indicating that greater material thickness did not further impair biocompatibility under the tested conditions (Fig. 3; Table 1).

**Table 1:**
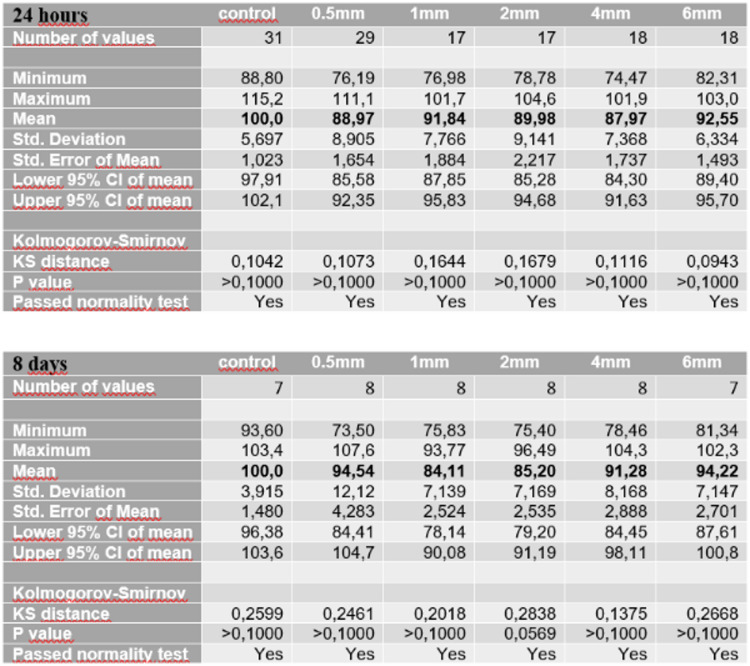
Descriptive statistics and normality test.

**Fig. 4:**
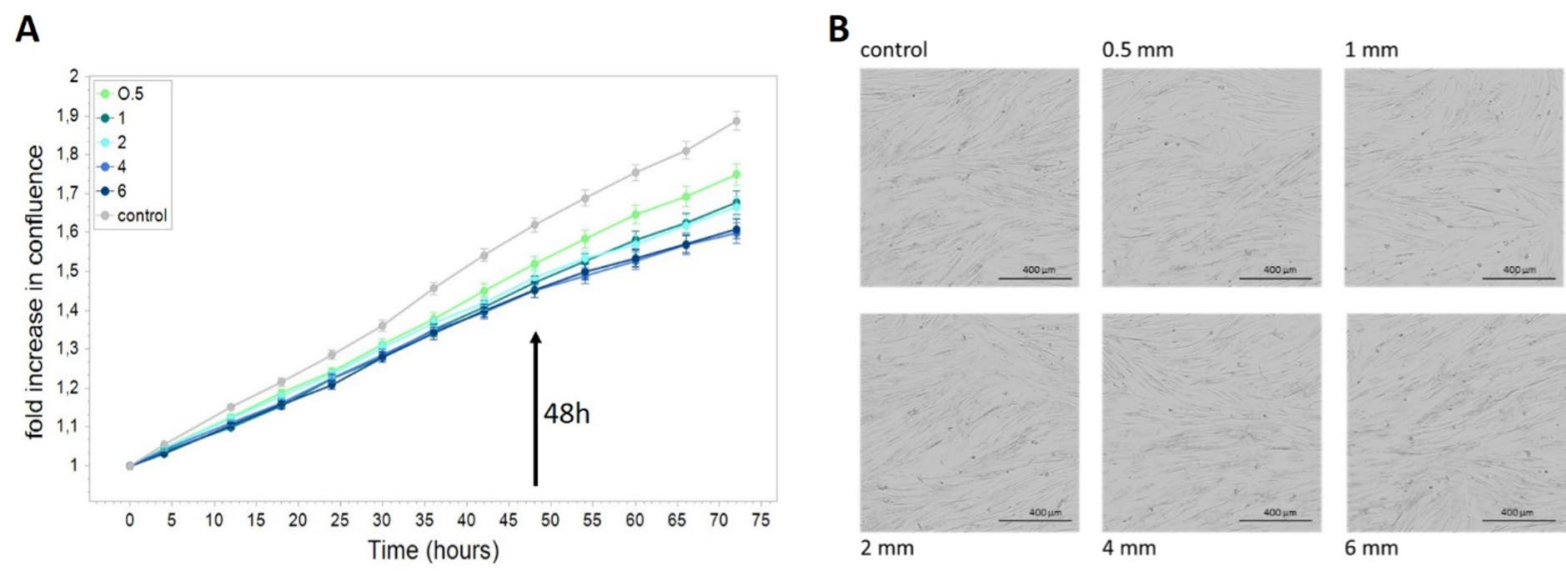
**A** Proliferation of HGF in media conditioned with LuxCreo test specimens (0.5, 1, 2, 4, 6 mm), monitored over time as fold increase in confluence by life cell imaging **B** HGFs in phase contrast (10x) at time point 48 h. The proliferation of HGFs treated with media conditioned with test specimens for 8 days shows no difference compared with the 24-hour results presented here.

Cell proliferation and morphology of human gingival fibroblasts (HGFs) were not significantly affected by the presence of specimens at any tested thickness. Proliferation curves obtained by live-cell imaging showed comparable growth dynamics across all thickness groups (Fig. 4A). Microscopic inspection confirmed that cell morphology remained normal, with no increase in rounded or detached cells (Fig. 4B).

## Discussion

Directly printed aligners (DPAs) are widely regarded as a promising advancement in clear aligner therapy compared with conventional thermoformed aligners, that have represented the clinical benchmark for almost three decades^6,23^. This originates due to DPA’s unique biomechanical and digital advantages, including the ability to selectively increase material thickness in specific areas to facilitate tooth movement while minimizing unwanted side effects, thereby enhancing overall treatment efficiency^8,10,12–17^. Beyond conventional aligner therapy, 3D printing also enables the easy and quick in-office fabrication of fully customized removable orthodontic appliances, such as the Twin-Block, which also require considerable increases in material thickness - an innovation that is widely regarded as a major breakthrough in modern digital orthodontics^8,10^.

In recent years, several companies have launched, or are currently in the process of developing, aligner resins for DPA fabrication^8–11^. This development reflects the rapidly expanding possibilities offered by digital design and 3D printing^7,23^. At the same time, it aligns with the growing trend among clinicians to reduce dependence on external aligner manufacturers and to work more autonomously. Furthermore, direct 3D printing of aligners reduces material waste, a feature that resonates strongly with both patients and practitioners in the context of increasing environmental awareness^24,25^. These benefits, together with enhanced biomechanical properties that improve treatment efficiency, reliability, and reduce unwanted side effects, have prompted pioneering and leading orthodontists worldwide to incorporate DPAs into their clinical practice, ranging from simple relapse cases to complex malocclusions^1,7,8,10,12–17^.

The ability to strategically design pressure and anchorage zones by increasing layer thickness, a key advantage of DPAs, has the potential not only to enhance biomechanics but also to reduce the number of necessary attachments, which in turn reduces chairside time and improves aesthetics^10,12^. Relevant clinical trade-offs, including attachment bonding performance and potential effects on enamel after removal, also need to be considered, as bonding efficacy depends on the aligner substrate and surface conditioning protocol^26^. However, this benefit of designing different layer thicknesses depends on high dimensional accuracy in the 3D printing process. Deviations in material thickness may affect key mechanical properties of the DPAs, such as resistance to deformation, stiffness, tensile strength, elastic modulus and stress relaxation^27,28^. Recently, Bleilöb et al. showed high accuracy in specimens made from Tera Harz TA-28 aligner resin (Graphy Inc, Seoul, South Korea) using the Asiga MAX 3D printer (Asiga SPS technology, Sydney, Australia)^10^. However, previous research investigating DPAs made from earlier materials, such as Tera Harz TC-85 and other resins, demonstrated that the manufactured DPAs tend to be thicker than their corresponding STL files^27,29,30^. Beyond the potential improvements in material properties offered by Tera Harz TA-28 (Graphy Inc, Seoul, South Korea) as well as LuxCreo DCA resin (LuxCreo Inc., Chicago, IL, USA), it is important to consider that the entire DPA manufacturing process involves multiple steps and a wide variety of different devices to perform them, depending on the aligner resin used^17^. The LuxAlign system (LuxCreo Inc., Chicago, IL, USA) addresses this potential source of inaccuracy by offering a strict end-to-end workflow^11^. This may represent a significant advantage over other systems, as it increases consistancy and reliability of the final DPAs.

To date, most available research has focused on DPAs made from Graphy’s original Tera Harz TC-85 aligner resin (Graphy Inc, Seoul, South Korea), with the primary emphasis placed on biomechanical performance rather than biocompatibility: Among the few available studies addressing biocompatibility, Pratsinis et al. reported that eluates from DPAs manufactured with Tera Harz TC-85 (Graphy Inc, Seoul, South Korea) exhibited no cytotoxic effects on human gingival fibroblasts, findings that are consistent with our results for DPA fabrication using LuxCreo’s DCA aligner resin (LuxCreo Inc., Chicago, IL, USA) as well as Tera Harz TA-28 (Graphy Inc, Seoul, South Korea)^10,19^. Furthermore, DPAs made from Tera Harz TC-85 (Graphy Inc, Seoul, South Korea) did not induce the proliferation of estrogen-sensitive MCF-7 cells^19^. However, considerable variability in UDMA release from DPAs fabricated with Tera Harz TC-85 (Graphy Inc, Seoul, South Korea) was identified by Willi et al., which may pose a potential health risk for patients^21^.

The evaluation of DPA biocompatibility at thicknesses greater than the standard 0.5 mm, as already performed by Bleilöb et al. for Tera Harz TA-28 (Graphy Inc, Seoul, South Korea), is of particular importance given the extensive design flexibility enabled by digital workflows and 3D printing^10^. Clinicians increasingly exploit the option to adapt aligner thickness to incresase the efficiancy of tooth movements or to design customized functional appliances such as Twin Block designs^8^. Notably, it is these functional appliances that are likely to benefit the most, as they can be fabricated far more rapidly and cost-effectively than with conventional techniques. Thus, research on the effect of the material thickness on the biocompatibility is very important for orthodontists using DPAs in their daily practice. This is particularly important, as the orthodontist bears responsibility for ensuring safe clinical use, both from a clinical and a legal perspective^10^.

To our knowledge, the present study is the first to assess both the biocompatibility and accuracy of DPAs manufactured from LuxCreo’s aligner DCA resin (LuxCreo Inc., Chicago, IL, USA) using the LuxAlign system (LuxCreo Inc., Chicago, IL, USA) and demonstrates that biocompatibility is maintained even at increased material thicknesses, provided that UV curing is performed according to the manufacturer’s post-processing guidelines. Increasing the material thickness up to 6 mm had no significant impact and therefore does not require any modification of the standard post-processing protocol. The standard post-processing protocol, including 20 min of UV curing, appears sufficient to ensure biocompatibility under the tested in vitro conditions. Although cell viability was reduced compared with the control, values remained above the 70% ISO 10993-5 threshold for cytotoxicity, and no statistically significant differences were observed between groups. However, further in vivo studies are recommended to determine whether this sub-cytotoxic reduction in viability may have any clinical relevance^31^. This appears particularly important, as previous studies on Graphy’s Tera Harz TC-85 and TA-28 (Graphy Inc, Seoul, South Korea) have shown considerable differences in reported cytotoxicity outcomes across published studies^31^. Based on previous research involving extended post-curing times of up to 60 min, and given that sufficient biocompatibility was achieved in the present study after 20 min of UV curing according to the manufacturer’s standard post-processing protocol, the effect of extended curing was not further investigated^10,20^.

When assessing the biocompatibility of DPAs, including those manufactured with Graphy’s TC-85 as well as TA-28 aligner resins (Graphy Inc., Seoul, South Korea), it must always be taken into account that both the software and hardware employed (e.g. the 3D printer used) may substantially influence not only biocompatibility but also accuracy and biomechanical properties of the final aligner^25^. LuxCreo’s LuxAlign system (LuxCreo Inc., Chicago, IL, USA) addresses this potential source of inconsistency by offering a fully and strictly integrated end-to-end workflow that does not permit substitution of any intermediate steps with alternative software or hardware from other manufacturers. Nevertheless, it must always be considered that production, and particularly post-processing, is a highly delicate process that requires careful and strict adherence. This is of particular importance as successful treatment outcomes rely on strict adherence to wearing times of 22 + hours a day during active treatment, with DPAs being exchanged every 7 to 14 days depending on the treatment protocol^10,21^. This means that the DPAs stay in constant contact with the oral mucosa and the initial high release rate of residual substances from newly inserted DPAs is maintained with each aligner change^20^.

Although this is an in-vitro study, which is limited in its ability to fully reproduce all intraoral influences such as chewing forces and bruxism, which may expose inner layers that might not be fully cured by damaging the aligners, as well as temperature fluctuations, pH variations, microbial activity, enzymatic reactions, aging effects, and cumulative exposure, we believe our results provide a reliable recommendation regarding whether prolongued UV curing is required when manufacturing DPAs with increased layer thickness to secure biocompatibility^10,19–21^. Nevertheless, it should be considered that the biocompatibility assessment in this study was limited to indirect cytotoxicity testing using human gingival fibroblasts. Allergy pathways, irritation or broader ISO 10993 biological endpoints that are important for long-term intraoral contact were not investigated. No artificial mechanical or thermal cycling protocols were applied, no enzymatic degradation processes were specifically simulated, and no chemical leachate or substance release analysis was performed. Thus, the specific compounds potentially released from the specimens over time could not be identified or quantified. These key limitations should be addressed in future research projects. Overall, the present findings provide a solid foundation for future investigations that can further explore the aspects mentioned above.

When evaluating the accuracy of the specimen discs, it should be considered that manual caliper measurements provide only limited information on overall dimensional accuracy and do not capture local thickness variations or internal surface deviations.

## Conclusions

DPAs represent a highly anticipated and significant advancement in clear aligner orthodontics. The present study demonstrated the high accuracy of the LuxAlign (LuxCreo Inc., Chicago, IL, USA) production process. Furthermore, the manufacturer’s standard production protocol, involving a 20-minute UV curing step during post-processing, appears to be sufficient, even for material thicknesses of up to 6 mm. These findings, however, require confirmation by further long-term an in vivo studies.

## Data Availability

All datasets are available upon request from the corresponding author.

## References

[1] Castroflorio, T., Parrini, S. & Rossini, G. Aligner biomechanics: Where we are now and where we are heading for. *J. World Fed. Orthod.* **13** (2), 57–64. 10.1016/j.ejwf.2023.12.005 (2024). 38228450 10.1016/j.ejwf.2023.12.005

[2] Weir, T. Clear aligners in orthodontic treatment. *Aust Dent. J.* **62** (Suppl 1), 58–62. 10.1111/adj.12480 (2017). 28297094 10.1111/adj.12480

[3] Rosvall, M. D., Fields, H. W., Ziuchkovski, J., Rosenstiel, S. F. & Johnston, W. M. Attractiveness, acceptability, and value of orthodontic appliances. *Am. J. Orthod. Dentofac. Orthop.* **135** (3), 276e1–27612. 10.1016/j.ajodo.2008.09.020 (2009). 10.1016/j.ajodo.2008.09.020 19268820

[4] Abbate, G. M. et al. Periodontal health in teenagers treated with removable aligners and fixed orthodontic appliances. *J. Orofac. Orthop.* **76** (3), 240–250. 10.1007/s00056-015-0285-5 (2015). 25929710 10.1007/s00056-015-0285-5

[5] Shokeen, B. et al. The impact of fixed orthodontic appliances and clear aligners on the oral microbiome and the association with clinical parameters: A longitudinal comparative study. *Am. J. Orthod. Dentofac. Orthop.* **161** (5), e475–e485. 10.1016/j.ajodo.2021.10.015 (2022). 10.1016/j.ajodo.2021.10.015 35248417

[6] Alkhamees, A. The new additive era of orthodontics: 3D-printed aligners and shape memory polymers-the latest trend-and their environmental implications. *J. Orthod. Sci.* **13**, 55. 10.4103/jos.jos_211_23 (2024). PMID: 39758107; PMCID: PMC11698253. 39758107 10.4103/jos.jos_211_23 PMC11698253

[7] Bichu, Y. et al. Advances in orthodontic clear aligner materials. *Bioactive Mater.* **10/20**10.1016/j.bioactmat.2022.10.006 (2022). 10.1016/j.bioactmat.2022.10.006 PMC9588987 36311049

[8] Ludwig, B., Ojima, K., Schmid, J. Q., Knode, V. & Nanda, R. Direct-printed aligners: A clinical status report. *J. Clin. Orthod.* **58** (11), 658–668 (2024). PMID: 39799597. 39799597

[9] Tera Harz Clear. doi:https://www.accessdata.fda.gov/cdrh_docs/pdf24/K240597.pdf

[10] Bleilöb, M., Welte-Jzyk, C., Knode, V., Ludwig, B. & Erbe, C. Biocompatibility of variable thicknesses of a novel directly printed aligner in orthodontics. *Sci. Rep.* **15** (1), 3279. 10.1038/s41598-025-85359-7 (2025). PMID: 39863636; PMCID: PMC11762277. 39863636 10.1038/s41598-025-85359-7 PMC11762277

[11] https://luxcreo.com/products/materials/dental-resins/dca/?utm_source=chatgpt.com

[12] Grant, J. et al. Forces and moments generated by 3D direct printed clear aligners of varying labial and lingual thicknesses during lingual movement of maxillary central incisor: an in vitro study. *Prog Orthod.* **24** (1), 23. 10.1186/s40510-023-00475-2 (2023). 37423974 10.1186/s40510-023-00475-2 PMC10329968

[13] Koenig, N. et al. Comparison of dimensional accuracy between direct-printed and thermoformed aligners. *Korean J. Orthod.* **52** (4), 249–257. 10.4041/kjod21.269 (2022). 35466087 10.4041/kjod21.269 PMC9314211

[14] Hertan, E., McCray, J., Bankhead, B. & Kim, K. B. Force profile assessment of direct-printed aligners versus thermoformed aligners and the effects of non-engaged surface patterns. *Prog. Orthodont.* **23** (1), 49. 10.1186/s40510-022-00443-2 (2022). 10.1186/s40510-022-00443-2 PMC9705625 36443390

[15] Lee, S. Y. et al. Thermo-mechanical properties of 3D printed photocurable shape memory resin for clear aligners. *Sci. Rep.* **12** (1), 6246. 10.1038/s41598-022-09831-4 (2022). 35428796 10.1038/s41598-022-09831-4 PMC9012852

[16] Can, E. et al. In-house 3D-printed aligners: effect of in vivo ageing on mechanical properties. *Eur. J. Orthod.* **44** (1), 51–55. 10.1093/ejo/cjab022 (2022). 33950232 10.1093/ejo/cjab022

[17] Zinelis, S., Panayi, N., Polychronis, G., Papageorgiou, S. N. & Eliades, T. Comparative analysis of mechanical properties of orthodontic aligners produced by different contemporary 3D printers. *Orthod. Craniofac. Res.* **25** (3), 336–341. 10.1111/ocr.12537 (2022). 34569692 10.1111/ocr.12537 PMC9544566

[18] Alessandra, C. et al. Comparison of the cytotoxicity of 3D-printed aligners using different post-curing procedures: an in vitro study. *Australasian Orthodontic J.* **39** (2), 49–56. 10.2478/aoj-2023-0026 (2023).

[19] Pratsinis, H. et al. Cytotoxicity and estrogenicity of a novel 3-dimensional printed orthodontic aligner. *Am. J. Orthod. Dentofac. Orthop.* **162** (3), e116–e122. 10.1016/j.ajodo.2022.06.014 (2022). 10.1016/j.ajodo.2022.06.014 35842359

[20] Iodice, G. et al. Effect of post-printing curing time on cytotoxicity of direct printed aligners: A pilot study. *Orthod. Craniofac. Res.*10.1111/ocr.12819 (2024). 38800926 10.1111/ocr.12819

[21] Willi, A. et al. Leaching from a 3D-printed aligner resin. *Eur. J. Orthod.* **45** (3), 244–249. 10.1093/ejo/cjac056 (2023). 36130120 10.1093/ejo/cjac056

[22] Narongdej P, Hassanpour M, Alterman N, Rawlins-Buchanan F, Barjasteh E. Advancements in Clear Aligner Fabrication: A Comprehensive Review of Direct-3D Printing Technologies. Polymers (Basel). **16** (3), 371. 10.3390/polym16030371. PMID: 38337260; PMCID: PMC10856925. (2024). 10.3390/polym16030371 PMC10856925 38337260

[23] Tartaglia, G. M. et al. Direct 3D Printing of Clear Orthodontic Aligners: Current State and Future Possibilities. *Mater. (Basel)*. **14** (7). 10.3390/ma14071799 (2021). 10.3390/ma14071799 PMC8038630 33916462

[24] Panayi, N., Cha, J. Y. & Kim, K. B. 3D Printed Aligners: Material Science, Workflow and Clinical Applications. *Semin. Orthod.* **29** (1), 25–33. (2023). 10.1053/j.sodo.2022.12.007

[25] Erbe, C., Ludwig, B. & Bleilöb, M. Unlocking the biological insights of 3D printed aligners: a look at current findings. *Semin Orthod.* **31**, 139–143. 10.1053/j.sodo.2024.10.001 (2025).

[26] Nguyen, V. A., Hoang, V., Vuong, T. Q. T., Phung, T. N. & Bich Hoang, N. P. Bond strength of thermoformed and 3D-printed aligners with universal primer versus one-step aligner adhesive with and without sandblasting: An in vitro study. *PLoS One*. **21** (1), e0341664. 10.1371/journal.pone.0341664 (2026). PMID: 41576052; PMCID: PMC12829832. 41576052 10.1371/journal.pone.0341664 PMC12829832

[27] Park, S. Y. et al. Comparison of translucency, thickness, and gap width of thermoformed and 3D-printed clear aligners using micro-CT and spectrophotometer. *Sci. Rep.* **13** (1), 10921. 10.1038/s41598-023-36851-5 (2023). 37407694 10.1038/s41598-023-36851-5 PMC10322848

[28] Sayahpour, B. et al. Effects of intraoral aging on mechanical properties of directly printed aligners vs. thermoformed aligners: an in vivo prospective investigation. *Eur. J. Orthod.* **46** (1). 10.1093/ejo/cjad063 (2024). 10.1093/ejo/cjad063 37936263

[29] Shirey, N., Mendonca, G., Groth, C. & Kim-Berman, H. Comparison of mechanical properties of 3-dimensional printed and thermoformed orthodontic aligners. *Am. J. Orthod. Dentofac. Orthop.* **163** (5), 720–728. 10.1016/j.ajodo.2022.12.008 (2023). 10.1016/j.ajodo.2022.12.008 37142355

[30] Edelmann, A., English, J. D., Chen, S. J. & Kasper, F. K. Analysis of the thickness of 3-dimensional-printed orthodontic aligners. *Am. J. Orthod. Dentofac. Orthop.* **158** (5), e91–e98. 10.1016/j.ajodo.2020.07.029 (2020). 10.1016/j.ajodo.2020.07.029 33131570

[31] Lorusso, M. et al. Cytotoxicity of Printed Aligners: A Systematic Review. *Dent. J. (Basel)*. **13** (7), 275. 10.3390/dj13070275 (2025). PMID: 40710120; PMCID: PMC12293704. 40710120 10.3390/dj13070275 PMC12293704

